# Longterm storage of post-packaged bread by controlling spoilage pathogens using *Lactobacillus fermentum* C14 isolated from homemade curd

**DOI:** 10.1371/journal.pone.0184020

**Published:** 2017-08-31

**Authors:** Soma Barman, Ranjan Ghosh, Shreya Sengupta, Narayan C. Mandal

**Affiliations:** 1 Mycology and Plant Pathology Laboratory, Department of Botany, Visva-Bharati, Santiniketan, West Bengal, India; 2 Heritage Institute of Technology, East Kolkata Township, Anandapur, West Bengal, India; Agricultural University of Athens, GREECE

## Abstract

One potent lactic acid bacterial strain C14 with strong antifungal activity was isolated from homemade curd. Based on morphological as well as biochemical characters and 16S rDNA sequence homology the strain was identified as *Lactobacillus fermentum*. It displayed a wide antimicrobial spectrum against both Gram-positive and Gram-negative pathogenic bacteria, and also against number of food spoilage, plant and human pathogenic fungi. The cell free supernatant (CFS) of the strain C14 was also effective against the fungi tested. Inhibition of radial growth of *Penicillium digitatum*, *Trichophyton rubrum* and *Mucor* sp. was noticed in the presence of CFS of C14 even at low concentration (1%). More than 94.3 ± 1.6% and 91.5 ± 2.2% inhibition of conidial germination of *P*. *digitatum* and *Mucor* sp. were noticed in the presence of 10-fold-concentrated CFS of C14. Massive deformation of the fungal mycelia was observed by SEM studies, and losses of cellular proteins and DNA are also evident upon its treatment with C14. HPLC analysis revealed the presence of phenyl lactic acid, lactic acid along with some unidentified compounds in the antifungal extract. Challenge experiment showed immense potential of the strain C14 in preventing the spoilage of bread samples caused by *Mucor* sp. and *Bacillus subtilis*. The bread samples remained fresh upto 25 days even after inoculation with *Mucor* sp. (3.7 × 10^4^ spores /ml) and *B*. *subtilis* (4.6 × 10^4^ CFU /ml). Along with the antifungal properties, the isolated lactic acid bacterial strain also showed very good antioxidant activities. Unchanged level of liver enzymes serum glutamic pyruvic transaminase and serum glutamic oxaloacetic transaminase in albino mice upon feeding with C14 also suggested non-toxic nature of the bacterial isolate.

## Introduction

Lactic acid bacteria (LAB) belonging to Gram positive group, are generally recognized as safe (GRAS) [1] and usually play an important role in food and feed fermentations as well as in their preservations. *Lactobacillus* is one of the largest genus in this category with almost 80 species that are widely used in different food products including sauerkraut, beer, pickle, wine, juices, cheese, yogurt and sausage [2]. LAB are also used as starter cultures for the production of dairy, meat and vegetable fermentation products [3,4]. In addition to their fermentation properties, a large number of LAB strains exhibit very good antifungal and antibacterial activities. Therefore, they are used as biopreservatives [5]. *Lactobacillus casei* inhibits the growth of *A*. *parasiticus* and its aflatoxin production [6]. *Lactobacillus fermentum* has strong antifungal properties against *Candida albicans* and *Candida glabrata* [7]. Apart from that, LAB are also able to produce bacteriocin like antibacterial compounds which are very much effective against numbers of pathogenic bacteria [8,9].

According to the Center for Science in the Public Interest (CSPI) various chemical preservatives cause serious health hazards to the mammalian system. Excess intake of sodium nitrate may cause tumor and cancer, salt form of sodium bicarbonate or baking soda increases blood pressure. Chemical preservatives like potassium bisulfate and potassium meta bisulfate exhibit allergic symptoms. On the other hand, LAB are quite safer and merely have any side effects on mammalian system.

Spoilage of food is a common phenomenon, generally caused by fungal growth which leads to economic loss. Excess fungal growth can also cause production of mycotoxins which is highly toxic to human and animals [10]. Among the different types of packaged food, spoilage of bread cause huge economic losses for bakery industry [11,12]. Several mould species belonging to *Aspergillus*, *Penicillium*, *Rhizopus* and *Mucor* are important organisms causing spoilage of bread. Apart from these, yeasts belonging to different genera also cause bread spoilage. Along with fungal and yeast contamination bacterial spoilage is also prevalent. Ropiness, which is the most significant spoilage of bread after mouldiness, occurs particularly under warm and humid conditions and is generally caused by *B*. *subtilis* [13]. During baking process of bread only vegetative cells are destroyed, while endospores of bacteria remain unharmed in bread [14]. The endospores of *Bacillus* spp. are thus transmitted to processed products where they could create several health problems of the consumers. Packaged bread can be protected from spoilage microorganisms by destroying the spores which contaminate the products, using LAB with broad antimicrobial spectrum [5].

The aim of the present study was to isolate and identify a food grade LAB strain from homemade curd for the prevention of spoilage pathogens. The antimicrobial activities against pathogenic bacteria, fungi and yeast as well as the probable mode of action of the antimicrobial compounds produced by the isolate have been assessed. Attempts have been taken to control the spoilage of post-packaged bread by controlling spoilage pathogens, thus to obtain more safe with an extended shelf life of bread.

## Materials and method

### Experimental design

**Fig 1:**
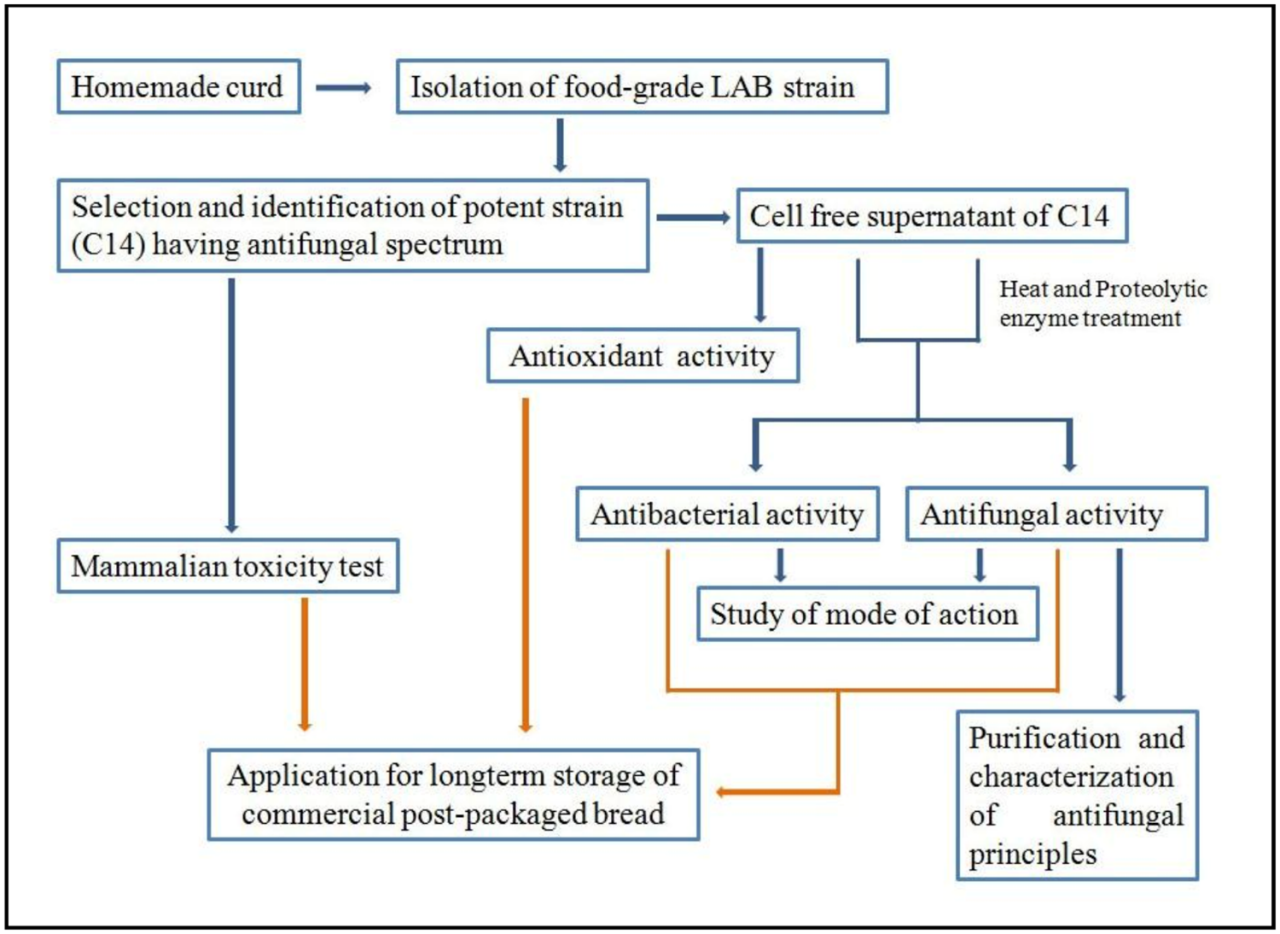
Schematic representation of the experimental design.

The general procedure started with isolation of potent LAB strains from homemade curd. Initially a number of LAB colonies were randomly taken to check their antimicrobial activities against *Candida albicans*. Finally one potent LAB strain was selected on the basis of its broad antifungal spectrum. The strain was identified by 16S rDNA sequence homology and by phylogenetic analysis. Production of antimicrobial metabolites in broth culture, effective against both fungal as well as bacterial pathogens was checked. The antimicrobial metabolites were subjected to heat and proteolytic enzyme treatments for testifying their nature. Modes of action of the antimicrobial compounds were studied against both bacterial and fungal pathogens. Antifungal principles were partially purified through DOWEX 50H^+^ column and characterized by HPLC. Finally an attempt was taken to control the spoilage of post-packaged commercial bread using this strain. The antioxidant potential of the bacterial metabolites was also studied for finding more value in them. For safety reasons, the mammalian toxicity of the strain was also examined. It was represented schematically in Fig 1.

### Microorganisms and culture conditions

All the isolated LAB strains were cultured in *Lactobacillus* MRS medium [15] (HiMedia, Mumbai, India) and were maintained in 10% glycerol-skimmed milk at 4°C. Bacteriocin sensitive indicator strain *Enterococcus faecalis* MB1 was kindly provided by Prof. Bibek Ray, Wyoming University, USA. Pathogenic bacterial strains used in antimicrobial assay were obtained from Microbial Type Culture Collection (MTCC), IMTECH, Chandigarh, India and were maintained in nutrient agar medium (HiMedia, Mumbai, India) in slants at 4°C. Fungal strains, *viz*., *Mucor* sp. VBBM7, *Rhizopus stolonifer* VBAM1 and *Penicillium digitatum* VBCS1 were isolated in our laboratory from spoiled bread samples, rotten surfaces of jackfruit and spoiled oranges respectively. Other fungal strains were also obtained from MTCC and were maintained in respective media as suggested by MTCC.

### Isolation of LAB with antifungal potential

The LAB strains were isolated from homemade curd samples on *Lactobacillus* MRS [15] (HiMedia, Mumbai, India) agar plates after serial dilutions. Plates were incubated at 28°C for 48 h. Initially 125 colonies were taken randomly and were checked for their antifungal potential against *Candida albicans* by dual culture overlay method [16]. Colonies producing prominent zones of inhibition (≥8 mm) were selected.

After initial screening five bacterial strains *viz*., C2, C8, C11, C14 and C29 were selected and were used to check their antifungal spectrum against 14 different fungal species which included animal as well as plant pathogenic and food spoilage fungi. The antifungal activities of LAB strains were studied by dual culture overlay method [16]. The zones of inhibition were measured and compared.

### Characterization and identification of the strain

The isolated LAB strain C14 was characterized morphologically with the help of a light microscope after Gram staining and endospore staining. Carbohydrate utilization, antibiotic sensitivity profile and other biochemical tests were performed following manufacturer guidelines of HiMedia (Mumbai, India).

The strain was identified following 16S rDNA sequence homology. The 16S region was amplified using forward (5’-TGGAGAGTTTGATCATGGCTC-3’) and reverse (5’- ACGGCTACCTTGTTACGACTT-3’) primers. Forward and reverse DNA sequencing reactions were carried out on ABI 3730xl Genetic Analyzer. Nucleotide sequence, generated from forward and reverse sequences was used to carry out BLAST analysis in NCBI GenBank database. A neighbor-joining phylogenetic tree [17] on the basis of 16S rDNA sequences was constructed following Kimura’s two parameter model [18] using MEGA5 [19]. *Lactococcus lactis* subsp. *lactis* JCM5805^T^ was taken as an out group member during phylogenetic analysis.

### Co-production of antimicrobial compounds in broth culture

Secretion of antimicrobial principle(s) to the extracellular broth by the strain C14 was checked by agar well diffusion method [20]. The appropriate solid media were used for bacteria and fungi. 50 μl of MRS culture aliquot, cell free supernatant (CFS) of the culture and heat killed supernatant were applied in different wells. For antibacterial study the pH of culture aliquot, CFS and heat killed supernatant were measured and adjusted to pH 6.8 ± 0.2. Plates were allowed to diffuse the antimicrobial principle(s) for 5 h at room temperature, incubated at 28°C and 37°C (for human pathogenic microorganisms) for visual growth of each microorganism and zones of inhibition were observed and diameters were measured.

### Effect of proteolytic enzymes on CFS of the strain

Effects of proteolytic enzymes, *viz*., proteinase K and trypsin (200 AU /ml) on filter sterilized CFS of C14 (adjusted to pH 6.8 ± 0.2) were tested after addition and incubation at 37°C for 30 min. Following the incubation both the antibacterial and antifungal potential of treated CFS were tested by agar well diffusion method [20].

### Study of mode of action of antimicrobial compound(s)

#### Study of bacterial growth curve

In order to check the mode of action of CFS of C14 on *B*. *subtilis* MTCC121 and *P*. *aeruginosa* MTCC741, growth pattern of the pathogens in the presence and in the absence of CFS of C14 were studied. 1% 24 h grown culture of each bacterium was added to 10 ml nutrient broth and incubated at their respective growth temperature. At the mid log phase of the growth 1 ml of 10-fold concentrated CFS (adjusted to pH 6.8 ± 0.2) of C14 was added to both bacterial cultures [9]. In control set only 10-fold concentrated uninoculated MRS broth was added. Properly diluted culture aliquots of 100 μl from each set were spread after proper dilution on nutrient agar plates at intervals of 1.5 h and CFUs were counted.

#### Study of potassium (K^+^) ions loss from bacterial cells

In order to determine the loss of K^+^ ions from bacterial cells, *B*. *subtilis* and *P*. *aeruginosa* cells were grown in 100 ml nutrient broth (pH 7.0) and harvested by centrifugation at 6,000 rpm for 10 min. Cells were re-suspended in 10 ml of 50 mM K-HEPES buffer (pH 6.5). 1 ml of 10-fold concentrated CFS (adjusted to pH 6.8 ± 0.2) of C14 was added to the cell suspensions of both the bacteria and incubated at room temperature for 20 min. Simultaneously, another two sets were prepared where both of the bacterial cells were treated with valinomycin (Sigma, USA) at a final concentration of 2 nM. All the sets of both bacterial strains were centrifuged (10,000 × g for 15 min) and the supernatants collected were filtered sterilized (0.22 μm filters) (Sartorius AG, Goettingen, Germany). The amount of K+ ion concentration of each filtrate was measured by atomic absorption spectrophotometer (Shimadzu, AA-6800, Japan).

#### Radial growth assay

The inhibitory activities of CFS of C14, against *Trichophyton rubrum*, *Penicillium digitatum* and *Mucor* sp. were studied by using poison food technique [21]. The CFS of C14 was mixed at a serial concentration of 1%~10% (vol/vol) with fungal media during plate preparations. The percentages of growth inhibition (I) were calculated, using the following formula: I (%) = [(C-T)/C] × 100. The corrected inhibitions were determined by IC (%) = [(C-T)/(C-C_0_)] × 100 where C is the control diameter, T is the test diameter and C_0_ is the diameter of test fungi agar discs (0.5 cm).

#### Conidial germination assay

The antifungal activity of the CFS of C14 was determined by conidial germination assay [22] on cavity slides. 10 μl of spore suspension containing about 3.7 × 10^4^ spores / ml in sterilized water was mixed with a solution of 190 μl 10-fold-concentrated CFS. The conidial suspension along with 10-fold-concentrated MRS broth was used as control. Sodium benzoate and calcium propionate (at a concentration of 3 mg /ml each) were considered as positive control. All the sets were incubated overnight at 28°C. Spore germinations were observed under light microscope and percentages of germination were calculated. Survivability of the treated spores was also checked on malt extract agar plates after long time incubation at 28°C.

#### SEM Study

To evaluate the effect of C14 on *Mucor* sp., *Trichophyton rubrum* and *Penicillium digitatum* scanning electron microscopic (SEM) studies of both treated and untreated mycelia were carried out. The mycelia were taken from inhibited zone interface as treated sample and from the uninhibited area as controlled one. They were prefixed with 2% glutaraldehyde (Merck Milipore) plus 5% dimethyl sulphoxide (DMSO) (Merck, Germany) for 30 min. followed by post-fixed with osmium tetraoxide (Sigma-Aldrich) and dehydrated in 30%-100% ethyl alcohol grades retaining them for 10 min in every dilution. The dehydrated mycelia were gold coated using an ion sputter (Coater IB-2, Gike engineering, Japan) and examined under SEM (HITACHI S-530, Japan) [23].

### Extraction and characterization of antifungal principle(s) from solid medium

C14 was inoculated as 2 cm long lines on MRS agar plates containing 1.2% agar agar (HiMedia, Mumbai) and incubated for 48 h at 28°C. The medium around the bacterial growth was scraped and added in sterilized water at the ratio of 1:10. Then the mixture was homogenized in a laboratory blender for 5 min and then vortexed for 10 min and was kept for 60 min at room temperature. The fluid obtained was transferred to vials for centrifugation at 5000 rpm for 10 min. Supernatants were filtered through 0.22 μm syringe filter, passed through DOWEX 50H^+^ (200–400 mesh) column [24] and lyophilized.

The lyophilisate was analyzed by HPLC analysis. For HPLC, standard solutions of lactic acid and phenyl lactic acid were included in the study. The sample was filtered through 0.22 μm syringe filter (Merck, Germany) before HPLC analysis. The analysis was performed on a C-18 column (250 × 4.6 mm, Phenomenex, Torrence, California, US) at room temperature. 20 mM H_3_PO_4_ at a flow rate of 1.0 ml /min was used as mobile phase. The eluate was monitored with prominence HPLC photo diode array detector (SPD-M20A, Shimadzu, Japan) at 210 nm. Data acquisition and management were completed with the help of LC Solution software.

### Study of fungal protein and nucleic acid loss

*Mucor* sp. and *T*. *rubrum* were grown for 48 h in 250 ml broth and were filtered to get fungal mycelia. Mycelia were washed with sterilized 50 mM sodium phosphate buffer followed by re-suspension in 25 ml of the same buffer. Mycelia of each fungus were treated with the 5 ml of antifungal extract (500 μg/ml) up to 10 h and the supernatants were withdrawn at intervals of two hours, centrifuged and the presence of nucleic acids and proteins were determined by measuring the optical densities at 260 nm and 280 nm respectively [25].

### Control of spoilage of post-packaged commercial bread using C14

The antifungal activities of C14 was checked by *in vitro* challenge study to control the spoilage of bread samples caused by *Mucor* sp. VBBM7 and *Bacillus subtilis* MTCC121. C14 was grown in 500 ml Erlenmeyer flask for 48 h and the cells were harvested by centrifugation at 6000 rpm for 10 min. The cells were resuspended in 5 mM sodium phosphate buffer at concentration of 5.8 × 10^7^ CFU /ml. Spore suspension of *Mucor* sp. was also prepared in sterilized water (3.7 × 10^4^ spores /ml). To destroy the vegetative cells only, liquid culture of *B*. *subtilis* was heated at 80°C for 15 min so that only spores were left. Finally, 4.6 × 10^4^ spores / ml of *B*. *subtilis* were taken for challenge experiment. Bread samples were cut into small pieces (4.0 ± 0.3 cm / 8.2 ± 0.4 cm), sterilized in petriplates and were divided into seven sets. The first set was treated only with C14 cell suspension. The second and the third sets were separately inoculated with *Mucor* sp. (3.7 × 10^4^ spores /ml) and *B*. *subtilis* (4.6 × 10^4^spores/ml) respectively. In the fourth, the fifth and the sixth sets C14 cell suspension was added before treatment with *Mucor* sp., *B*. *subtilis* separately and together respectively. A seventh set was kept where bread pieces were not treated with any organisms. All the bread samples were kept at room temperature (26.0 ± 2.0°C) up to 30 days and incidence of bread spoilages were observed.

To check the physical presence of LAB on the bread surfaces, C14 was re-isolated from treated bread samples after 30 days of incubation. One gram of bread was crushed in distilled water to a final volume of 10 ml, serially diluted and plated on MRS medium containing norfloxacin (50 μg / ml) as an antibiotic marker.

### Antioxidant activity

Antioxidant activity of metabolites produced by C14 was determined using DPPH (Sigma-Aldrich) assay [26]. The 10-fold-concentrated CFS of C14 was dissolved in methanol at a concentration of 10 mg/ml. 100 μl of various concentrations of the methanolic fractions were mixed with 2900 μl DPPH solution and incubated in darkness at room temperature for 30 min. The absorbance was recorded at 517 nm. The percentage of free radical scavenging activity of methanolic extract was calculated as: %I = [(A_blank_-A_sample_)/A_blank_] ×100 Where A_blank_: Absorbance of control; A_sample_: Absorbance of methanolic extract and DPPH. IC_50_ was calculated by plotting straight line equation.

### Animal experiment

#### Ethics statement

Eight—ten weeks old male mice weighing about 20–25 g were taken in animal experiment. Mice were kept at two mice per stainless steel community cages with a 12 h light and dark cycle in a temperature controlled room (25 ± 2°C). Mice were acclimatized for about one week prior to treatment. Mice showing any abnormal behavior were immediately removed from the cage. Animal experiment was performed in accordance with the guidelines of the Committee on Institutional Animal Care and Use to ensure the wellbeing of the animals. The experiment was approved by the Chairperson of the Animal Ethics Committee, Visva-Bharati, Santiniketan (S1, S2 and S3 Files).

#### Mammalian toxicity test

To check any kind of toxicity of the isolated LAB C14 on mammalian system, bacterial suspension 5.8 × 10^7^ CFU / ml was administered to albino mice for a month through drinking water in respective feeding bottles. Fresh bacterial cultures were administered in every day feeding. The mice were provided with standard mouse diet (NMC Oil Mills Ltd., Pune, India). The sample size of mice was n = 4. After one month of treatment, blood samples were collected through cardiac puncture where Titriplex^®^ III (Merck Milipore) was used as an anticoagulant. SGPT and SGOT levels of collected blood samples were measured by using a transaminase assay kit (505-0P; Sigma, St. Louis, Mo.) according to the manufacturer's instructions. All surgical experiments were performed under ketamine (Sigma-Aldrich) anesthesia and efforts were made to minimize suffering of the animals. Developments of any pathogenic lesion or inflammatory symptoms in albino mice due to live C14 feeding were also observed.

### Data analyses

All the experiments were repeated and replicated at least thrice and results were independently observed. The means and standard deviations were calculated using Microsoft Excel 2007 program.

## Results

### Isolation, characterization and identification of LAB

**Fig 2:**
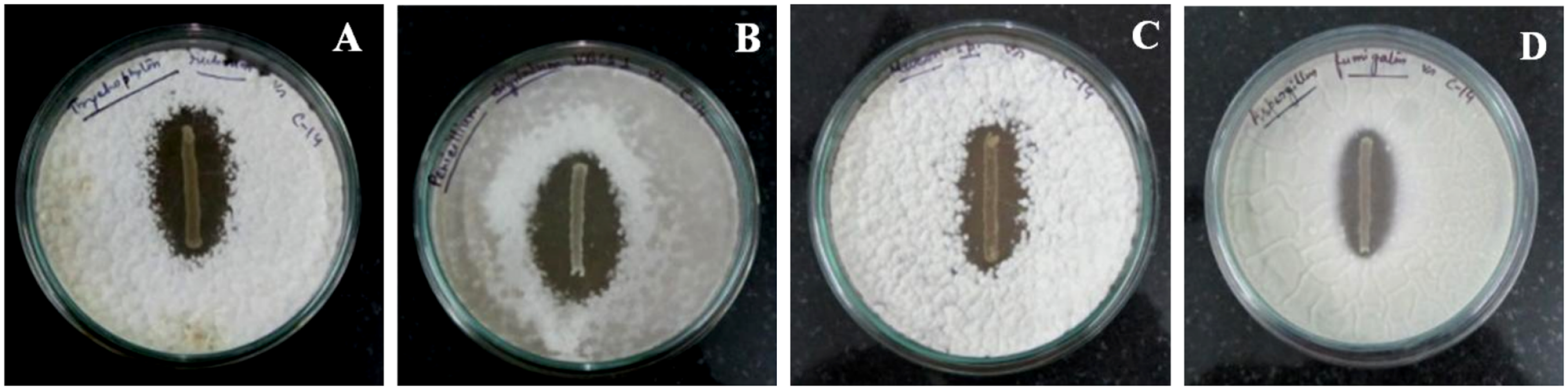
Zones of inhibition produced by C14 against pathogenic fungi *Trichophyton rubrum* MTCC296 (A), *Penicillium digitatum* VBCS1 (B), *Mucor* sp. VBBM7 (C), *Aspergillus fumigatus* MTCC2796 (D).

During isolation of LAB initially 125 colonies were randomly selected. Among them, C2, C8, C11, C14 and C29 strains showed antifungal activity against *Candida albicans* (Table 1). When the antifungal spectrum of these five isolates was checked by dual culture overlay method against a number of animal and plant pathogenic fungi, they were able to produce prominent zones of inhibition (Table 1). As the strain C14 showed quite large antifungal spectrum it was considered for further studies. It could produce zones of inhibitions (Fig 2) against 11 pathogenic fungal strains among 14 fungi tested (Table 1).

**Table 1:** 

Fungal strains	Zones of inhibition in cm ± SD				
	C2	C8	C11	C14	C29
*Penicillium digitatum* VBCS1	2.4 ± 0.2	3.1 ± 0.3	2.9 ± 0.1	3.4 ± 0.2	2.8 ± 0.3
*Colletotrichum acutatum* MTCC1037	3.5 ± 0.3	2.9 ± 0.2	3.3 ± 0.1	3.6 ± 0.4	2.8 ± 0.3
*Rhizopus stolonifer* VBAM1	2.2 ± 0.2	2.3 ± 0.1	2.2 ± 0.4	2.5 ± 0.2	1.9 ± 0.3
*Aspergillus parasitcus* MTCC2976	1.8 ± 0.4	1.9 ± 0.2	2.5 ± 0.1	3.3 ± 0.4	-
*Aspergillus fumigatus* MTCC2550	-	-	1.6 ± 0.5	3.1 ± 0.1	2.1 ± 0.3
*Mucor* sp. VBBM7	2.9 ± 0.2	2.5 ± 0.3	2.7 ± 0.1	3.2 ± 0.2	1.7 ± 0.4
*Candida albicans* MTCC183	1.6 ± 0.2	1.4 ± 0.3	1.9 ± 0.2	2.3 ± 0.3	1.8 ± 0.2
*Curvularia lunata* MTCC2098	-	-	-	-	-
*Cladosporium* sp. MTCC351	-	-	-	-	-
*Microsporum gypseum* MTCC2819	-	-	-	4.2 ± 0.3	-
*Trichophyton rubrum* MTCC296	-	-	-	3.2 ± 0.2	-
*Alternaria alternata* VBAV007	1.7 ± 0.4	1.9 ± 0.2	2.1 ± 0.3	3.3 ± 0.1	2.9 ± 0.2
*Aspergillus flavus* MTCC2799	-	-	-	2.3 ± 0.1	-
*Candida tropicalis* MTCC184	-	-	-	-	-

Antifungal activity of five LAB isolates against pathogenic fungi. (-) Indicates absence of inhibition zone, each values were measured by mean of breath of zones of inhibition.

**Fig 3:**
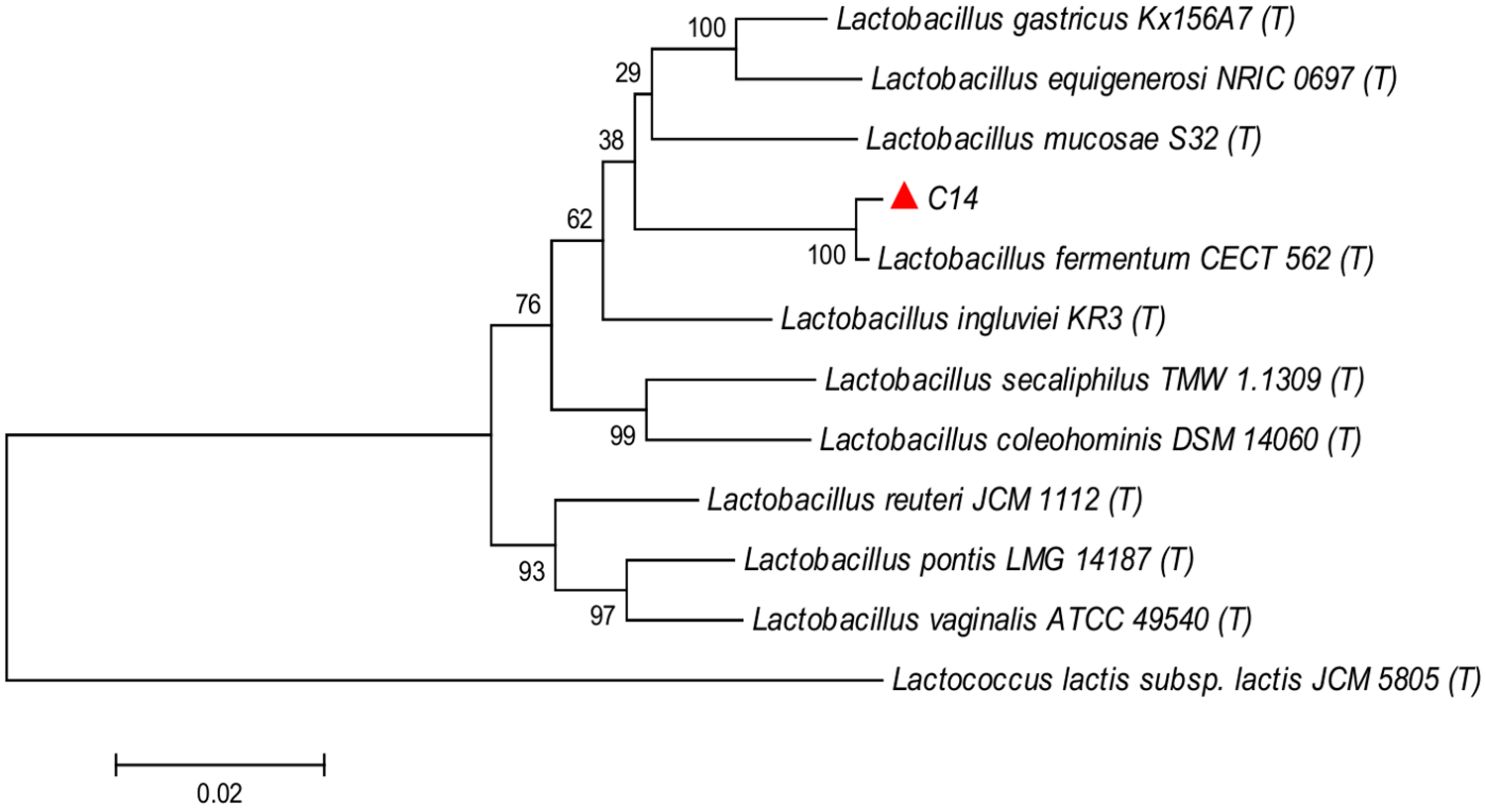
Neighbor joining phylogenetic tree based on 16S rDNA sequence of *Lactobacillus fermentum* C14 showing relationship with other members.

The strain C14 produced small white colonies on *Lactobacillus* MRS agar plates. It was found to be Gram positive, non-motile, non-endospore forming rod present singly or in pairs. It showed positive reaction for nitrate reductase test but negative for catalase, MR-VP and indole tests. The strain was able to utilize lactose, dextrose, maltose, fructose, sucrose and inulin when tested using Himedia carbohydrate kit (Himedia KB009). It also showed positive results for esculin hydrolysis test. Based on 16S rDNA sequence homology and phylogentic analysis (Fig 3) the strain C14 was identified as *Lactobacillus fermentum*. A nucleotide sequence of 1417 bp of *L*. *fermentum* C14 strain was generated from forward and reverse sequence data.

### Co-production of antimicrobial compounds in broth culture

The 24 h grown CFS of the strain showed pH 5.4 ± 0.2 before neutralization. Liquid culture as well as CFS of C14 has showed antifungal activities by producing zones of inhibition. Loss of antifungal activity after boiling the CFS was also noticed except in *Mucor* sp. and *P*. *digitatum*.

On the other hand both boiled and unboiled neutralized (pH 6.8 ± 0.2) CFS of C14 was able to produce prominent zones of inhibition both against Gram positive as well as Gram negative pathogenic bacteria (Table 2). Proteolytic enzyme assay with proteinase K and trypsin showed loss of antibacterial activity in the CFS but antifungal activity was retained after this treatment.

**Table 2:** 

Bacteria	Zones of Inhibition (cm) ± SD		
	Liquid culture	CFS	Heat killed CFS
*Enterococcus faecalis* MB1	1.3 ± 0.3	1.2 ± 0.15	1.2 ± 0.1
*Bacillus subtilis* MTCC121	1.6 ± 0.02	1.5 ± 0.1	1.5 ± 0.15
*Listeria monocytogenes* MTCC657	1.0 ±0.3	1.0 ± 0.2	1.0 ± 0.2
*Staphylococcus aureus* MTCC96	0.9 ± 0.3	0.9 ± 0.25	0.9 ± 0.25
*Pseudomonas aeruginosa* MTCC741	1.2 ± 0.15	1.1 ± 0.1	1.1 ± 0.1
*Salmonella typhimurium* MTCC98	1.1 ± 0.15	1.0 ± 0.15	0.9 ± 0.1
*Pantoea ananatis* MTCC2309	1.1 ± 0.2	1.0 ± 0.2	1.0 ± 0.15
*Escherichia coli* MTCC1667	-	-	-

Antibacterial activity of *Lactobacillus fermentum* C14 against different pathogenic bacteria. (-) Indicates absence of inhibition zone.

### Mode of action of antibacterial compound

**Fig 4:**
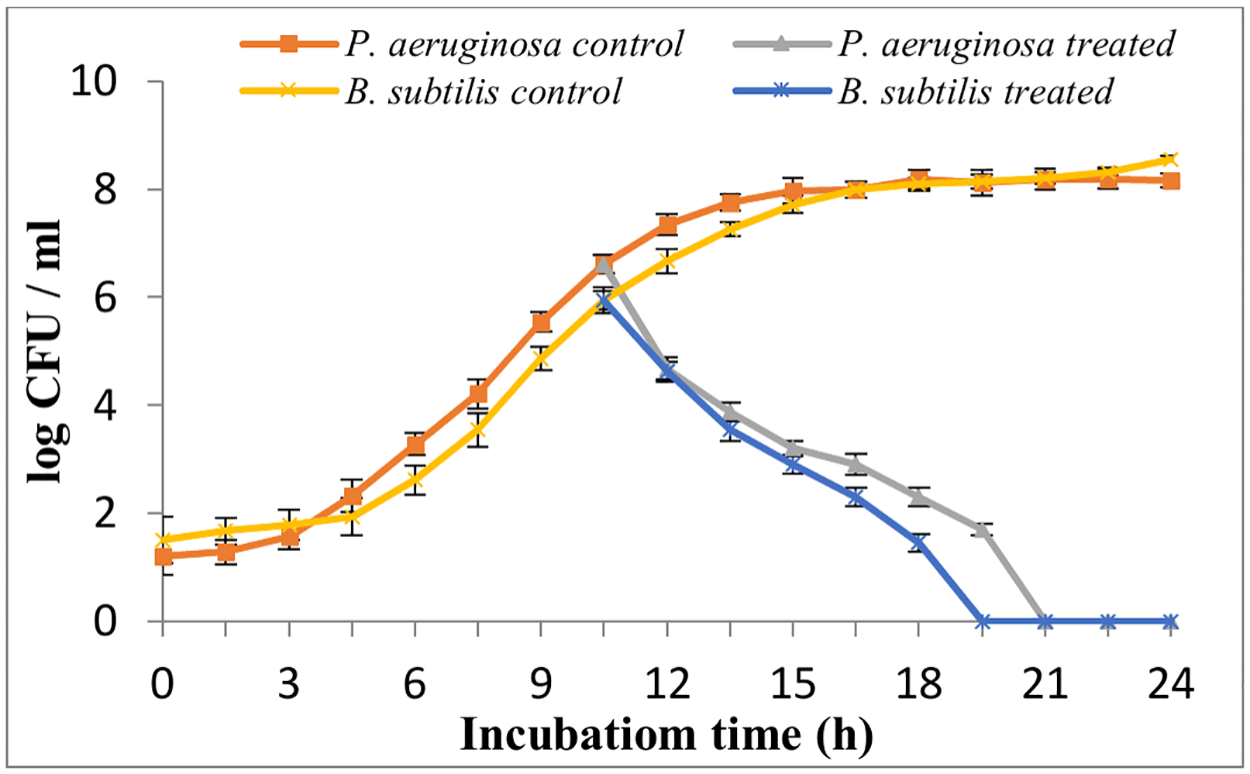
Effect of cell free supernatant of C14 on the growth of *B*. *subtilis* MTCC121 and *P*. *aeruginosa* MTCC741. The results were the mean of triplicate trials.

To determine the mode of action of antibacterial compound on *B*. *subtilis* and *P*. *aeruginosa*, growth curve patterns were studied in the presence and absence of CFS of C14. The results showed a rapid decline in the CFU count in both the tested bacteria immediately after the addition of the CFS and no viable counts after 10 h of addition (Fig 4).

Loss of K^+^ ion from CFS treated cells of *B*. *subtilis* and *P*. *aeruginosa* were noticed. The average K^+^ ion concentrations of untreated *B*. *subtilis* and *P*. *aeruginosa* cells were 0.34 ± 0.08 ppm and 0.28 ± 0.13 ppm respectively, whereas K^+^ ion concentration of ten-fold concentrated CFS treated *B*. *subtilis* and *P*. *aeruginosa* and were 6.52 ± 0.35 ppm and 5.98 ± 0.26 ppm and valinomycin (2 nM) treated cells were 4.56 ± 0.92 ppm and 3.89 ± 1.02 ppm respectively.

### Inhibition of radial growth and conidial germination of pathogenic fungi

**Fig 5:**
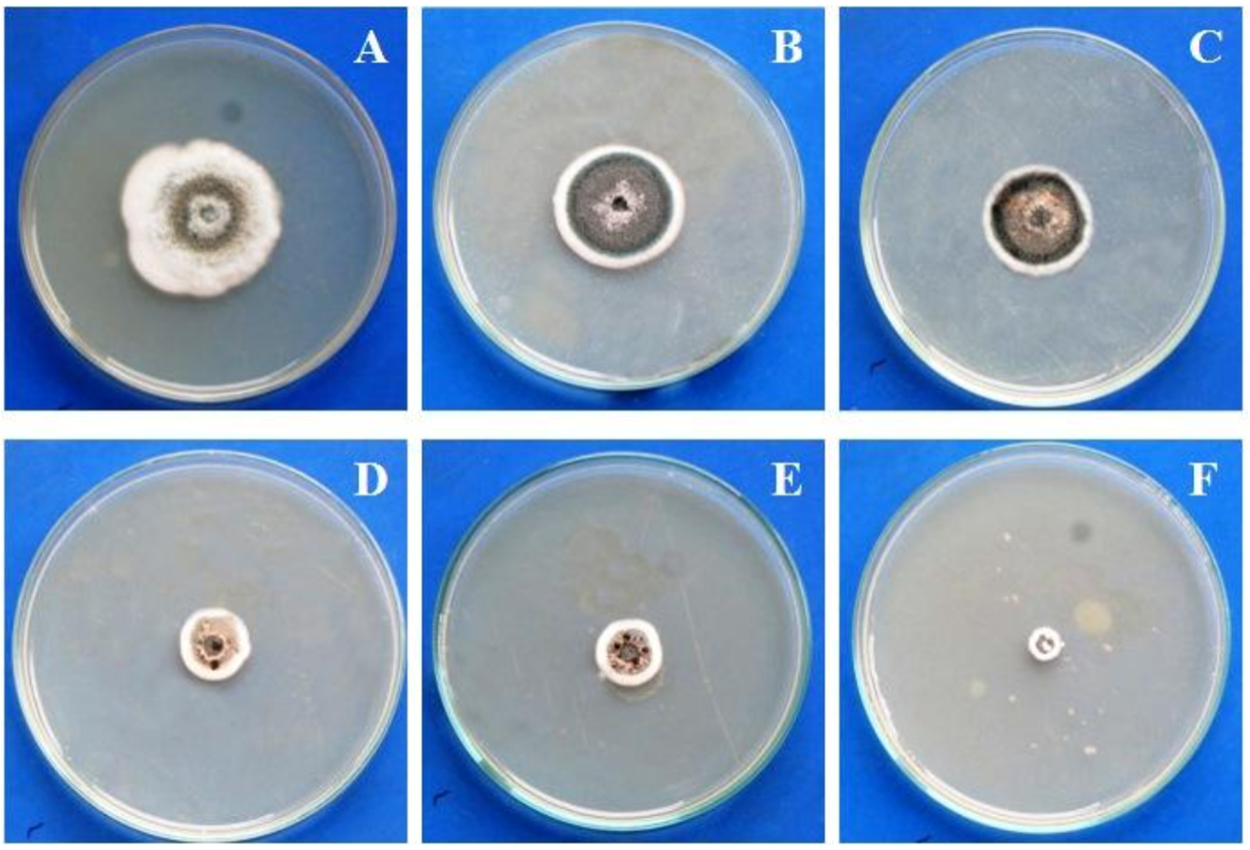
Inhibition of radial growth of *Trichophyton rubrum* MTCC296 by cell free supernatant of C14; control (A), treated with cell free supernatant of C14 at different concentrations (B-F) (B- 1%; C-3%; D-5%; E-7%; F-10%).

During radial growth assay different concentrations of CFS of C14 were used against *Trichophyton rubrum*, *Penicillium digitatum* and *Mucor* sp. The CFS was highly effective in controlling the radial growth (Fig 5) of all the three fungi. Growth inhibition of *Trichophyton rubrum* to the extent of 73.6 ± 2.1% were noticed in the presence of 10% CFS of C14, whereas 92.5 ± 1.8% and 86.3 ± 2.7% growth inhibitions of *P*. *digitatum* and *Mucor* sp. were observed in the presence of 10% CFS respectively (Table 3).

**Table 3:** 

Sample concentration (%)	Corrected growth inhibition IC (%) ± SD		
	*T*. *rubrum*	*P*. *digitatum*	*Mucor* sp.
1%	33.4 ± 2.6	27.5 ± 2.0	22.6 ± 1.6
3%	48 ± 1.9	50 ± 3.1	41.3 ± 2.8
5%	53.5 ± 2.4	72.5 ± 2.6	64.6 ±3.2
7%	66.7 ± 1.9	77.5 ± 2.2	75.4 ± 2.1
10%	73.6 ± 2.1	92.5 ± 1.8	86.3 ±.2.7

Inhibition of radial growth of pathogenic fungi by CFS of *Lactobacillus fermentum* C14.

The CFS of C14 was also found to be effective to inhibit the conidial germination of *Penicillium digitatum* as well as *Mucor* sp. *in vitro*. More than 94.3 ± 1.6% and 91.5 ± 2.2% inhibitions of conidial germination of *P*. *digitatum* and *Mucor* sp. were noticed in the presence of 10-fold-concentrated CFS. More than 48% inhibition of conidial germination was observed for both of the fungi even in the presence of 2-fold-concentrated CFS (Table 4). The activity of *L*. *fermentum* C14 was found to be fungicidal after treatment with CFS of *L*. *fermentum*, and the conidia did not survive after prolonged incubation in malt extract medium at 28°C.

**Table 4:** 

	Mean Percentages (%) of inhibition ± SD			
Fungal species	Chemicals		*L*. *fermentum* C14	
	Calcium propionate (3 mg / ml)	Sodium benzoate (3 mg / ml)	2-fold conc. CFS	10-fold conc.CFS
*P*. *digitatum* VBCS1	18.6 ± 2.3	72.8 ± 1.6	50.4 ± 1.2	94.3 ± 1.6
*Mucor sp*.VBBM7	15.2 ± 0.8	64.7 ± 2.6	48.7 ± 0.5	91.5 ± 2.2

Inhibition of germination of conidia by chemicals and CFS of *L*. *fermentum* C14.

### Morphological disintegrations of pathogenic fungi by C14

**Fig 6:**
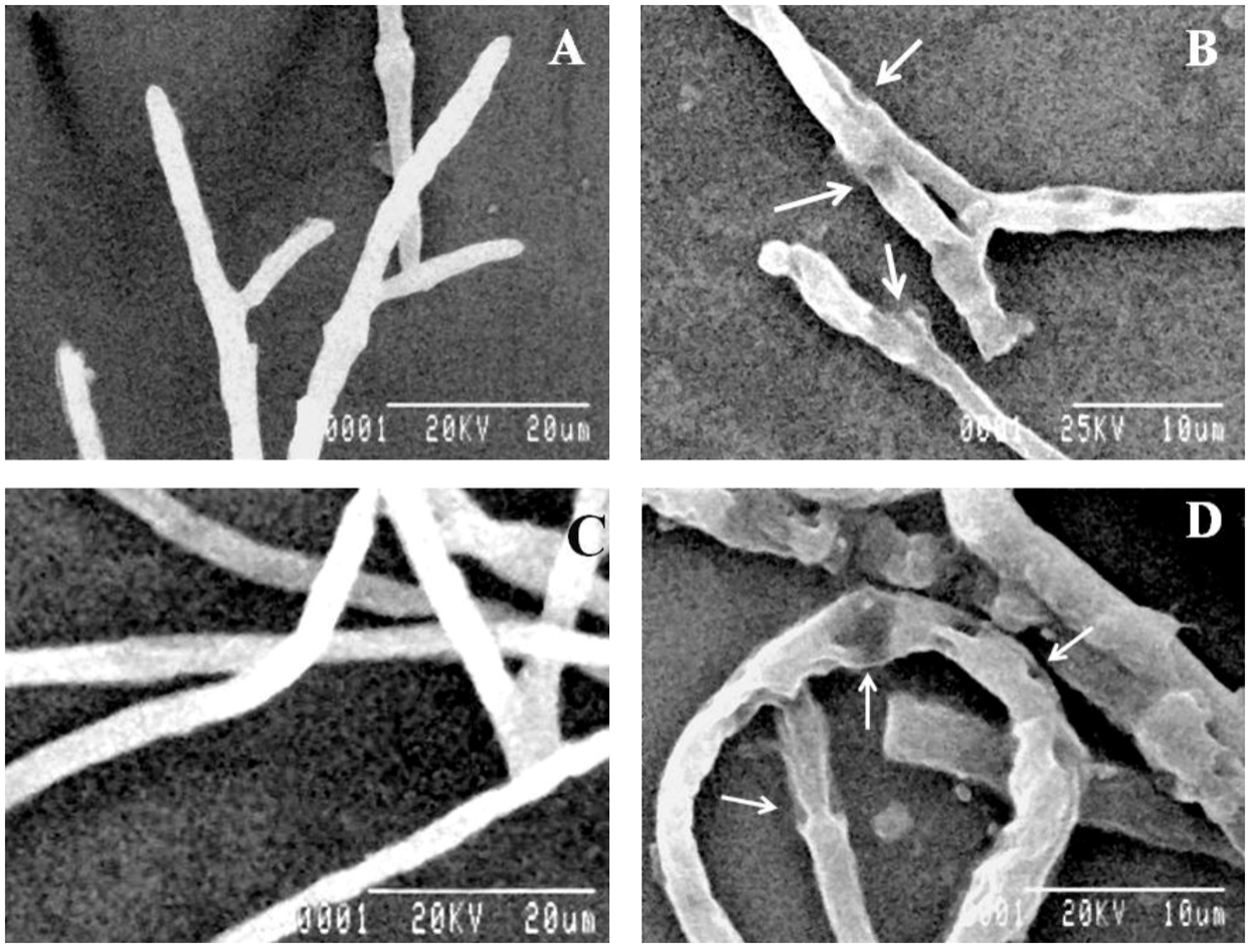
Scanning electron micrographs of fungal mycelia of *Mucor* sp. VBBM7 control (A), *Mucor* sp. VBBM7 treated with C14 (B), *Trichophyton rubrum* MTCC296 control (C), *T*. *rubrum* MTCC296 treated with C14 (D).

When C14 was cultured against the bread mould *Mucor* sp. VBBM7 (Fig 6B) or human pathogenic *T*. *rubrum* MTCC297 (Fig 6D), clear mycelia disintegrations were observed by SEM. Such type of morphological aberrations were absent in case of untreated controls (Fig 6A and 6C). The damages induced by the LAB strain were evidenced by the formation of pores on the hyphal surfaces or sometimes a massive degeneration or chewing of hyphal structures was also observed.

### Characterization of antifungal principles

**Fig 7:**
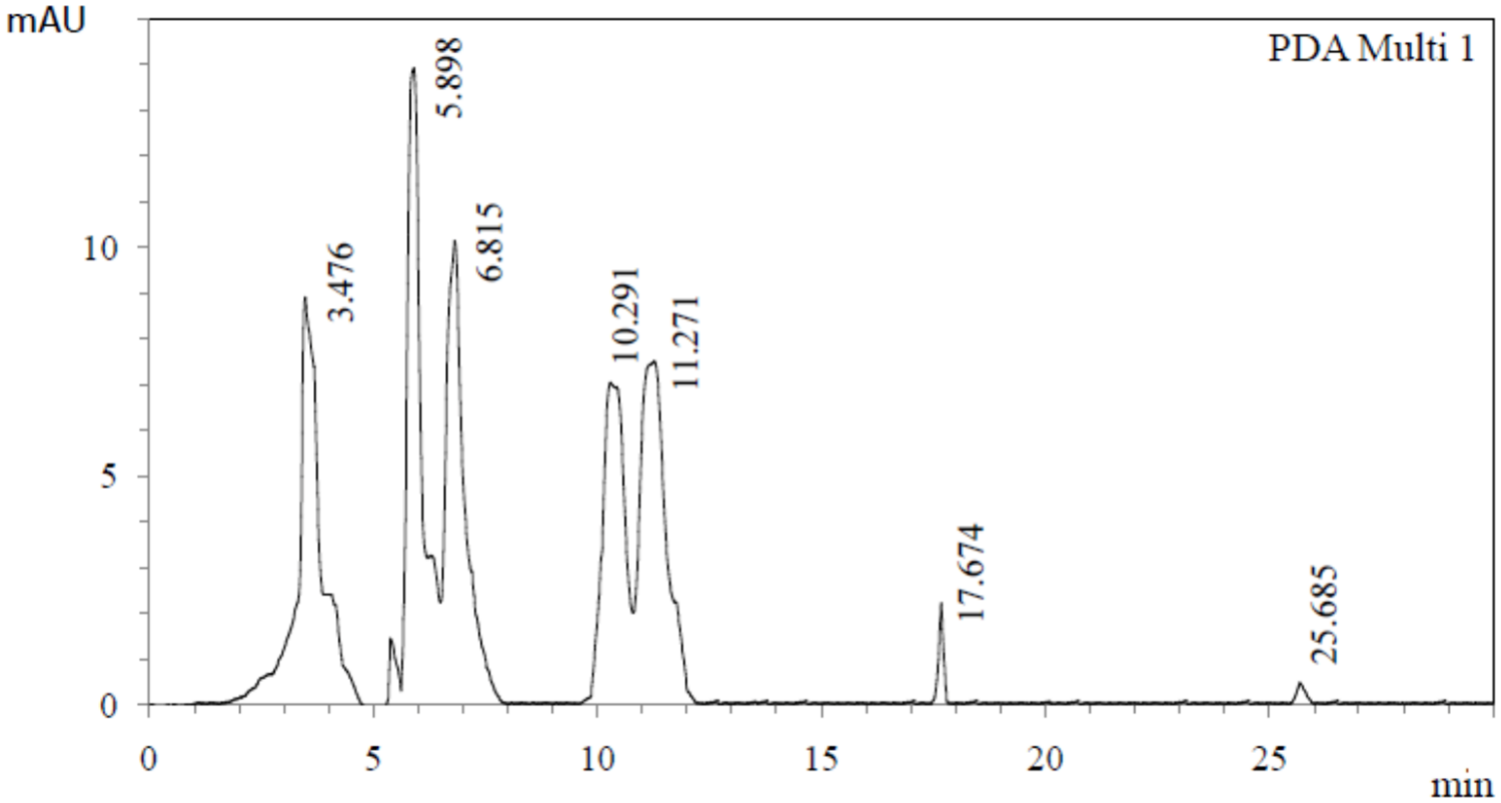
HPLC chromatogram of isolated antifungal compounds from culture broth of *L*. *fermentum* C14.

Presence of phenyl lactic acid and lactic acid in the extract was confirmed by comparing the data obtained from HPLC analysis using commercial phenyl lactic acid and lactic acid (Sigma, US) respectively. Prominent peaks for the presence of phenyl lactic acid and lactic acid were detected at the retention time of 3.476 min and 5.898 min respectively (Fig 7). Along with these two acids the presence of some unidentified peaks at the retention time of 6.815 min, 10.291 min and 11.271 min were also detected (Fig 7).

### Release of intra-cellular materials from fungal mycelia

When fungal mycelia of *Mucor* sp. and *T*. *rubrum* were treated with antifungal extracts efflux of intra-cellular proteins and nucleic acids were observed. Increase of protein and nucleic acid concentration in the buffer were recorded after 4 h and 6 h of treatments respectively.

### Control of spoilage of post-packaged commercial bread using C14

**Fig 8:**
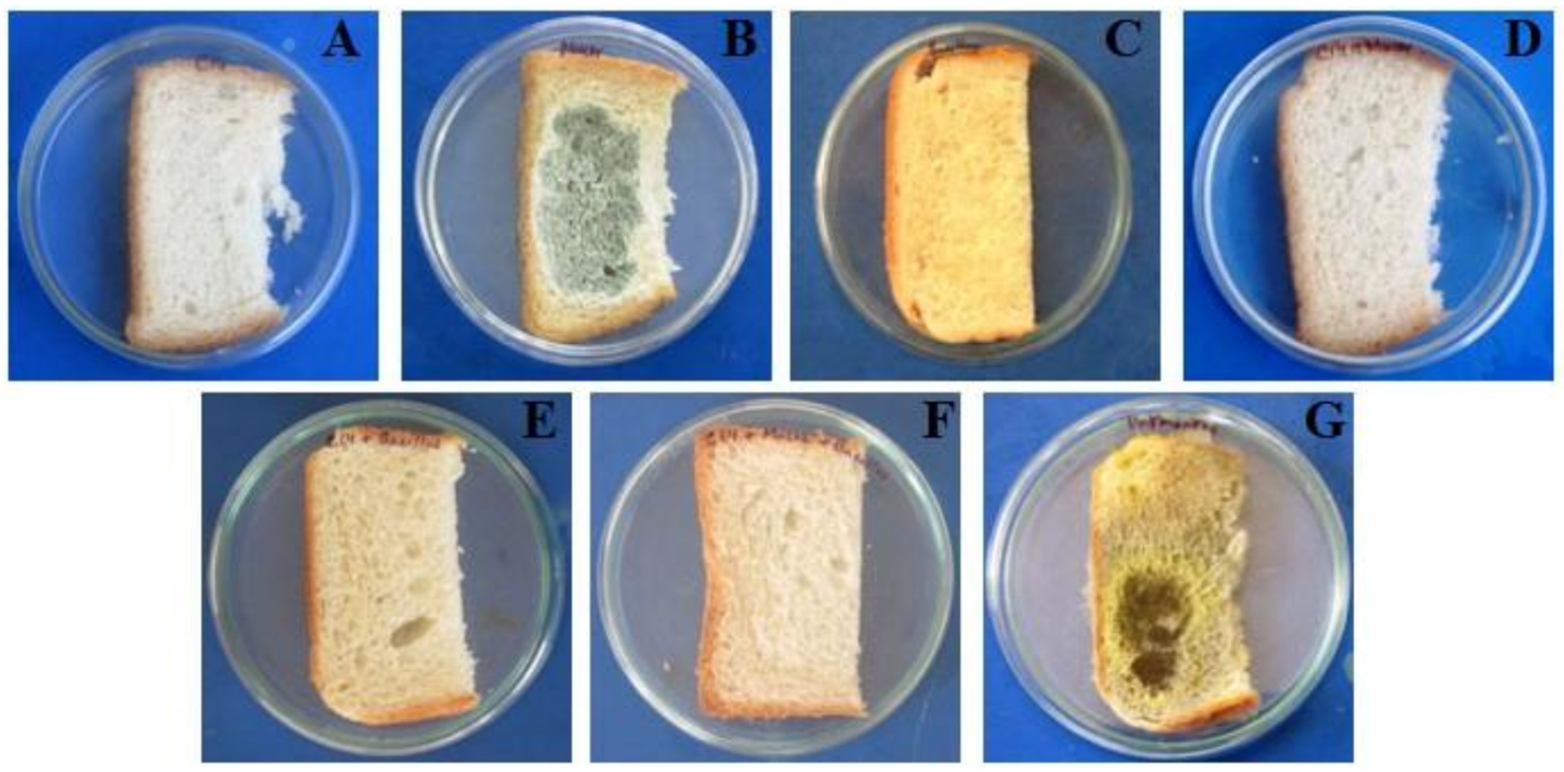
Control of spoilage of bread samples using *L*. *fermentum* C14; treated with C14 cell suspension (5.8 × 10^7^ CFU/ml) (A), treated with *Mucor* sp. spores (3.7 × 10^4^ spores/ml) (B), treated with *B*. *subtilis* (4.6 × 10^4^ spores /ml) (C), treated with C14 cell suspension (5.8 × 10^7^ CFU /ml) as well as *Mucor* sp. spores (3.7 × 10^4^ spores /ml) (D), treated with *B*. *subtilis* (4.6 × 10^4^ spores /ml) (E), treated with C14 cell suspension (5.8 × 10^7^ CFU /ml) as well as *B*. *subtilis* (4.6 × 10^4^ spores / ml) (F), untreated (G).

During the experiment, it was found that the bread samples of fourth, fifth and sixth sets remained fresh for more than 25 days where C14 had been applied before treatment with *Mucor* sp. and *B*. *subtilis* and a combination of both of them respectively (Fig 8D–8F). Massive fungal growth was observed in the control set within 4 days (Fig 8G) whereas spoilage of breads by *Mucor sp* and *B*. *subtilis* was noticed in the second (Fig 8B) and the third sets (Fig 8C) respectively within few days of incubation. No spoilage was noticed in case of the first set (Fig 8A) treated only with cell suspension of C14.

Survivability of the strain C14 was checked on bread surfaces. A considerable number of bacterial cells were found per g of bread samples even after 30 days of its application (Table 5).

**Table 5:** 

Experimental set	CFU in different days (per g of bread)			
	0	7	15	30
Set 4	3.3×10^7^	2.8×10^7^	3.6×10^6^	2.95×10^5^
Set 5	2.7×10^7^	1.9×10^7^	3.25×10^6^	2.4×10^5^
Set 6	3.1×10^7^	2.6×10^7^	3.05×10^6^	1.97×10^5^

Survivability of *Lactobacillus fermentum* C14 in bread at different days

### Antioxidant activity and mammalian toxicity of C14

Prominent color change of DPPH from pink to yellow indicated very good antioxidant property of the concentrated CFS. From the standard curve of percentage (%) of inhibitions, IC_50_ value of 10-fold concentrated CFS of C14 was calculated as 91.70.

No significant increase of SGPT and SGOT in the blood samples of treated mice indicated the non-toxic nature of the LAB isolate in the mammalian system. In addition no pathogenic or inflammatory symptoms were detected in treated mice after one month of treatment.

## Discussion

Among the different LAB isolates, five strains, *viz*., C2, C8, C11, C14 and C29 showed very good antifungal activities against a number of pathogenic fungi. Among them C14 was selected as prospective strain for further studies based on its best antifungal spectrum. The strain was identified by 16S rDNA sequence homology as *Lactobacillus fermentum*.

Antifungal activities of several LAB have already been reported by several workers. *Lactobacillus rhamnosus* L60 and *Lactobacillus fermentum* L23 showed antifungal activities against aflatoxin producing strain of *Aspergillus* sp. [27]. Presence of the antifungal as well as antibacterial principles in CFS indicated the secretion of antimicrobial compound(s) by C14 in the extracellular medium. Loss of antifungal activities after boiling also indicated the thermo-sensitive property of the antifungal principle(s). Mild inhibition of *Mucor* sp. and *Penicillium digitatum* by boiled CFS may be due to its decrease of pH. Production of phenyl lactic acid and 4-hydroxy phenyl lactic acid as antifungal compounds were already reported from *Lactobacillus plantarum* 21B isolated from sourdough [5]. Mandal *et al*. (2013) [23] reported the presence of an inducer dependent novel antifungal compound(s) by *Pediococcus acidilactici* LAB5, where they have found phenyl lactic acid as one of the active compounds. On the other hand the zones of inhibition produced by the boiled supernatant of C14 against pathogenic bacteria suggested thermo-stable nature of antibacterial compound produced by C14. Such type of bacteriocin like thermo-stable antimicrobial compound production by *Lactobacillus fermentum* has already been reported by several workers [8]. As the pH of the CFS were adjusted to 6.8 ± 0.2 during antibacterial assay, therefore antibacterial activity of C14 were not due to any acidic compounds. Loss of antibacterial potential upon protease treatments of the CFS of C14 and retention of antifungal activity under similar condition confirmed the proteinaceous antibacterial principle and non-protein antifungal compound(s) produced by the same organism respectively. Previous reports suggested that the antifungal activities of LAB are mainly dependent on the production of organic acids like lactic acid or phenyl lactic acids. Increase of pH of the CFS of LAB reduced the antifungal potential of these organic acids due to their dissociation [28,29]. Therefore in the present study the pH of the CFS were not neutralized during antifungal study. Further chemical characterization of the active principles would throw better light regarding their detailed mechanism of action.

To know the mode of action of the antibacterial thermostable CFS, C14 was added to mid log phase of *B*. *subtilis* MTCC121 and *P*. *aeruginosa* MTCC741. A rapid decline in CFU count of both the bacterial strains in treated set indicated the strong bacteriocidal nature of the antibacterial compound(s).

During radial growth assay, it was observed that CFS of C14 reduced the radial growth of *Trichophyton rubrum*, *Penicillium digitatum* and *Mucor* sp. very efficiently. *Trichophyton rubrum* is a serious human pathogenic fungus, causing severe skin infections, whereas *Penicillium digitatum* is a plant pathogen causing major post harvest spoilage of orange. On the other hand *Mucor* sp. is responsible for spoilage of bread and other food products. Inhibition of these fungal strains by the strain C14 suggested the broad field of application in bakery. Svanström *et al*. (2013) [30] also reported the inhibition of radial growth of *Aspergillus niger*, *Cladosporium gloeosporioides* and *Penicillium roqueforti* by phenyl lactic acid produced by LAB.

Drastic distortions as well as pore formations on fungal hyphae were also observed by SEM study upon treatment with C14. Such type of mycelial degradation of *Rhizopus stolonifer* VBAM1 by *Lactococcus lactis* subsp. *lactis* was reported previously [31].

Increase of protein and nucleic acid concentration in the extracellular buffer, upon treatment with antifungal compound(s), indicated cell wall breakage and membrane leakage of the fungal mycelia.

Lactic acid was detected as the major antifungal compound produced by C14 during HPLC analysis along with phenyl lactic acid and some unidentified compounds which might also be responsible for the antifungal activity of the strain. Production of lactic acid by *Lactobacillus fermentum* CRL220 and some peptide antifungal compounds by *Lactobacillus fermentum* CRL251 has been reported earlier [32].

Most interestingly, C14 was found to have the ability to control the growth of *Mucor* sp. and *B*. *subtilis* on bread surfaces very efficiently even when it was challenged with high numbers of fungal and bacterial spores. Cizeikiene *et al*. (2013) [33] also reported control of rope spoilage of bread samples caused by *B*. *subtilis* using starter culture of *Pediococcus pentosaceus* KTU05-9 upto 4 days at 30°C. Simultaneous control of endospore forming *B*. *subtilis* and fungal spoilage organism by the culture suspension of *L*. *fermentum* C14 is novel in this regard. The experiment indicated a co-production of antifungal as well as bacteriocin like antibacterial principle by the strain.

Survivability is an important factor for the organisms used as a preservative for longtime storage of the food product. Presence of appreciable number of bacterial count of C14 in bread even after 30 days of application, strongly suggested its positive role in successful longtime preservation of bread.

Oxidative damages have several pathological roles in human diseases causing cancer, cirrhosis, arthritis, emphysema etc. [34]. Although several synthetic antioxidants like butylated hydroxytoluene, butylated hydroxyanisole have strong antioxidant activities, but possess many side effects [35]. Antioxidants from natural sources are safer than synthetic products. Along with the antifungal properties C14 also showed very good antioxidant activity which provides an extra advantage for it as food preservative.

Most of the toxic compounds, when entered in the mammalian system, are either metabolized to its non-toxic forms in liver or are excreted out. In many occasions, liver cannot metabolize the toxic molecules which in turn damage liver-cells significantly resulting excessive release of SGOT and SGPT in blood. Therefore SGPT and SGOT, these two liver enzymes are used as significant markers of parenchymal liver damage caused by toxic substances [36]. Upon treatment with C14, the level of these two enzymes remained unchanged like untreated control set which indicated non-toxic nature of the isolates and supported its application for human consumption.

## Conclusion

*Lactobacillus fermentum* C14, isolated from homemade curd, showed very good antimicrobial activity against a number of plant as well as animal pathogenic fungi and bacteria. The CFS of the bacterial strain also showed effective results in controlling the growth of fungal and bacterial pathogens. It was capable of inhibiting the spoilage of post-package commercial bread, caused by *Mucor* sp. and also showed very good antioxidant activity. As LAB recommended by Food and Drug Administration (FDA), have GRAS status and the strain C14 showed no mammalian toxicity, therefore antimicrobial properties of *L*. *fermentum* C14 would have a promising application as biopreservative in bakery products in place of chemical preservatives.

## Supporting information

S1 File Animal experiment statement. (PDF)

S2 File ARRIVE Guideline Checklist. (PDF)

S3 File Animal ethics approval. (PDF)

## Abbreviations

AU: Activity unit
CFS: Cell free supernatant
CFU: Colony forming unit
DPPH-1: 1-Diphenyl-2-picrylhydrazyl
LAB: Lactic acid bacteria
MRS: de Man Rogosa and Sharpe
SEM: Scanning electron microscope
SGOT: Serum glutamic oxaloacetic transaminase
SGPT: Serum glutamic pyruvic transaminase

## References

[1] Avall-Jaaskelainen S, Palva A. *Lactobacillus* surface layers and their applications. FEMS Microbiol Rev. 2005; 29: 511–529. 15935509 10.1016/j.femsre.2005.04.003

[2] Prescott LM, Harley JP, Kelin DA. Microbiology, 5th ed Boston, New York; 2002.

[3] Konings WN, Kok J, Kuipers OP, Poolman B. Lactic acid bacteria: the bugs of the new millennium. Curr Opin Microbiol. 2000; 3: 276–282. . 10851157 10.1016/s1369-5274(00)00089-8

[4] De Vuyst L, Leroy F. Bacteriocins from Lactic Acid Bacteria: Production, Purification, and Food Applications. J Mol Microbiol Biotechnol. 2007; 13: 194–199. doi: 10.1159/000104752 17827969 10.1159/000104752

[5] Lavermicocca P, Valerio F, Evidente A, Lazzaroni S, Corsetti A, Gobbetti M. Purification and characterization of novel antifungal compounds from the sourdough *Lactobacillus plantarum* strain 21B. Appl Environ Microbiol. 2000; 66(9): 4084–4090. . 10966432 10.1128/aem.66.9.4084-4090.2000 PMC92262

[6] El-Gendy SM, Marth EH. Growth and aflatoxin production by *Aspergillus parasticus* in the presence of *Lactobacillus casei*. J Food Prot. 1981; 44: 211–212. 10.4315/0362-028X-44.3.211 30836487

[7] Masood M, Qadir MI, Shirazi JH, Khan IU. Beneficial effects of lactic acid bacteria on human beings. Crit Rev Microbiol. 2011; 37(1): 91–98. doi: 10.3109/1040841X.2010.536522 21162695 10.3109/1040841X.2010.536522

[8] Pascual LM, Daniele MB, Giordano W, Pájaro MC, Barberis IL. Purification and partial characterization of novel bacteriocin L23 produced by *Lactobacillus fermentum* L23. Curr Microbiol. 2008; 56(4): 397–402. doi: 10.1007/s00284-007-9094-4 18172715 10.1007/s00284-007-9094-4

[9] Barman S, Ghosh R, Mandal NC. Use of bacteriocin producing *Lactococcus lactis* subsp. *lactis* LABW4 to prevent *Listeria monocytogenes* induced spoilage of meat. FNS. 2014; 5: 2115–2123. doi: 10.4236/fns.2014.522224

[10] Sweeney M, Dobson A. Mycotoxin production by *Aspergillus*, *Fusarium* and *Penicillium* species. Int J Food Microbiol. 1998; 43: 141–158. 9801191 10.1016/s0168-1605(98)00112-3

[11] Thompson JM, Waites WM, Dodd CER, Detection of rope spoilage in bread caused by *Bacillus* species. J Appl Microbiol. 1998; 85: 481–486.

[12] Dewettinck K, Van Bockstaele F, Kuühne B, Van de Walle D, Courtens TM, Gellynck X, Nutritional value of bread: influence of processing, food interaction and consumer perception. J Cereal Sci. 2008; 48: 243–257.

[13] Valerio F, De Bellis P, Lonigro SL, Visconti A, Lavermicocca P, Use of *Lactobacillus plantarum* fermentation products in bread-making to prevent *Bacillus subtilis* ropy spoilage. Int J Food Microbiol. 2008; 122: 328–332. doi: 10.1016/j.ijfoodmicro.2008.01.005 18261817 10.1016/j.ijfoodmicro.2008.01.005

[14] Pepe O, Blaiotta G, Moschetti G, Greco T, Villani F, Rope-producing strains of *Bacillus* spp. from wheat bread and strategy for their control by lactic acid bacteria. Appl Environ Microbiol. 2003; 69(4): 2321–2329. doi: 10.1128/AEM.69.4.2321-2329.2003 12676716 10.1128/AEM.69.4.2321-2329.2003 PMC154770

[15] de Man JC, Rogosa M, Sharpe ME. A medium for the cultivation of lactobacilli. J Appl Bacteriol. 1960; 23: 130–135.

[16] Magnusson J, Schnürer J. *Lactobacillus coryniformis* subsp. *coryniformis* strain SI3 produces a broad-spectrum proteinaceous antifungal compound. Appl Environ Microbiol. 2001; 67: 1–5. doi: 10.1128/AEM.67.1.1-5.2001 11133421 10.1128/AEM.67.1.1-5.2001 PMC92504

[17] Saitou N, Nei M. The neighbor-joining method: A new method for reconstructing phylogenetic trees. Mol Biol Evol. 1987; 4: 406–425. doi: 10.1093/oxfordjournals.molbev.a040454 3447015 10.1093/oxfordjournals.molbev.a040454

[18] Kimura M. A simple method for estimating evolutionary rate of base substitutions through comparative studies of nucleotide sequences. J Mol Evol. 1980; 16: 111–120. 7463489 10.1007/BF01731581

[19] Tamura K, Peterson D, Peterson N, Stecher G, Nei M, Kumar S. MEGA5: Molecular Evolutionary Genetics Analysis using maximum likelihood, evolutionary distance, and maximum parsimony methods. Mol Biol Evol. 2011; 28(10): 2731–2739. doi: 10.1093/molbev/msr121 . 21546353 10.1093/molbev/msr121 PMC3203626

[20] Fernández-Garayzábal JF, Delgado C, Blanco M, Vázquez-Boland JA, Briones V, Suárez G, et al Role of potassium tellurite and brain heart infusion in expression of the hemolytic phenotype of *Listeria* spp. on agar plates. Appl Environ Microbiol. 1992; 58: 434–438. 1539991 10.1128/aem.58.1.434-438.1992 PMC195232

[21] Wang HK, Yan YH, Wang JM, Zhang HP, Qi1 W, Production and characterization of antifungal compounds produced by *Lactobacillus plantarum* IMAU10014. PLoS ONE. 2012; 7(1): e29452 doi: 10.1371/journal.pone.0029452 22276116 10.1371/journal.pone.0029452 PMC3261852

[22] Lavermicocca P, Iacobellis NS, Simmaco M, Graniti A. Biological properties and spectrum of activity of *Pseudomonas syringae* pv. *syringae* toxins. Physiol Mol Plant Pathol. 1997; 50: 129–140.

[23] Mandal V, Sen SK, Mandal NC. Production and partial characterisation of an inducer-dependent novel antifungal compound(s) by *Pediococcus acidilactici* LAB 5. J Sci Food Agr. 2013; 93: 2445–2453. doi: 10.1002/jsfa.6055 23423982 10.1002/jsfa.6055

[24] Suh JW, Lee SH, Chung BC. GC-MS Determination of organic acids with solvent extraction after cation-exchange chromatography. Mol Pathol Gen. 1997; 43(12): 2256–2261. . 9439441

[25] Mandal V, Sen SK, Mandal NC. Assessment of antibacterial activities of pediocin produced by *Pediococcus acidilactici* LAB5. J Food Safety. 2010; 30: 635–651. doi: 10.1111/j.1745-4565.2010.00230.x

[26] Blois MS. Antioxidant determinations by the use of a stable free radical. Nature. 1958; 181: 1199–1200. doi: 10.1038/1811199a0

[27] Gerbaldo GA, Barberis C, Pascual L, Dalcero A, Barberis L. Antifungal activity of two *Lactobacillus* strains with potential probiotic properties. FEMS Microbiol Lett. 2012; 332(1): 27–33. doi: 10.1111/j.1574-6968.2012.02570.x 22497448 10.1111/j.1574-6968.2012.02570.x

[28] De Muynck C, Leroy AIJ, De Maeseneire, Arnaut F, Soetaert W, Vandamme EJ, Potential of selected lactic acid bacteria to produce food compatible antifungal metabolites. Microbiol Res. 2004; 159: 339–345. doi: 10.1016/j.micres.2004.07.002 15646380 10.1016/j.micres.2004.07.002

[29] Li H, Liu L, Zhang S, Cui W, Lv J, Identification of antifungal compounds produced by *Lactobacillus casei* AST18. Curr Microbiol. 2012; 65: 156–161. doi: 10.1007/s00284-012-0135-2 22580887 10.1007/s00284-012-0135-2

[30] Svanström A, Boveri S, Boström E, Melin P. The lactic acid bacteria metabolite phenyl lactic acid inhibits both radial growth and sporulation of filamentous fungi. BMC Research Note. 2013; 6(464): 1–9. doi: 10.1186/1756-0500-6-464 24229396 10.1186/1756-0500-6-464 PMC3835548

[31] Ghosh R, Barman S, Mukhopadhyay A, Mandal NC. Biological control of fruit-rot of jackfruit by rhizobacteria and food grade lactic acid bacteria. Biol control. 2015; 83: 29–36. doi: 10.1016/j.biocontrol.2014.12.020

[32] Gerez CL, Torres MJ, Font de Valdez G, Rollán G. Control of spoilage fungi by lactic acid bacteria. Biol Control. 2013; 64: 231–237. doi: 10.1016/j.biocontrol.2012.10.009

[33] Cizeikiene D, Juodeikiene G, Paskevicius A, Bartkiene E. Antimicrobial activity of lactic acid bacteria against pathogenic and spoilage microorganism isolated from food and their control in wheat bread. Food Control. 2013; 31: 539–545.

[34] Halliwell B, Gutteridge JM. Oxygen toxicity, oxygen radicals, transition metals and disease. Biochem J. 1984; 219(1): 1–14. 6326753 10.1042/bj2190001 PMC1153442

[35] Abramovič H, Abram V. Effect of added rosemary extract on oxidative stability of *Camelina sativa* oil. Acta Agriculturae Slovenica. 2006; 87: 255–261.

[36] Al-Hamz MA, Assaggaf AI, Al-Sayed GNE, Bin-Naser YS. Effect of acute and subchronic administration of nutmeg seeds extract on mice behaviour, histological structure and biochemical functions. Saudi J Biol Sci. 2004; 11: 177–187.

