# Supplementary material for: Longterm storage of post-packaged bread by controlling spoilage pathogens using Lactobacillus fermentum C14 isolated from homemade curd
Source: PLoS One. 2017 Aug 31;12(8):e0184020. doi: 10.1371/journal.pone.0184020 (PMC5578496; doi:10.1371/journal.pone.0184020)
Supplement: S1 File — (PDF) [file pone.0184020.s001.pdf]

Title of work: Testing of possible toxicity in mice induced by lactic acid bacteria

One potent lactic acid bacterial strain C14 with strong antifungal activity was isolated from homemade curd. Based on morphological as well as biochemical characters and 16S rDNA sequence homology the strain was identified as *Lactobacillus fermentum*. It displayed a wide antimicrobial spectrum against both Gram-positive and Gram-negative pathogenic bacteria, and also against number of food spoilage, plant and human pathogenic fungi.

Objectives of the present study:

To check any kind of toxicity of *Lactobacillus fermentum* C14 in mammalian system. SGPT and SGOT, these two liver enzymes are used as significant markers of parenchymal liver damage caused by toxic substances.

Proposed source of animals:

Saha Enterprise (Regd No. 1828/PO/Bt/S/15/CPCSEA).

Place where the animals will be kept (or proposed to be kept):

Animal House Facility, Department of Zoology, Visva-Bharati.

Mode of treatment:

Bacterial suspension was administered to swiss albino mice for a month through drinking water in respective feeding bottles. The experimental set as follows:

Animals required:

a. Species / Common name: *Mus musculus* (Swiss albino mice)
b. Age/ weight/ size: 8-10 weeks/ 20-25gm
c. Gender: Male
d. Number to be used (Year-wise breakups and total figures needed to be given):
Total number of mice = $(4 \times 2) = 8) \times 3 = 24$.

After one month of treatment, blood samples will be collected through cardiac puncture. SGPT and SGOT levels of collected blood samples will be measured by using a transaminase assay kit (505-0P; Sigma, St. Louis, Mo.) according to the manufacturer's instructions.

Surgical experiments will be performed under ketamine (Sigma-Aldrich) anesthesia and efforts were made to minimize suffering of the animals.

Probable outcome:

If the applied bacterium would not show any kind of toxicity, the SGPT and SGOT levels would not increase in blood. So the applicability of the bacterium (*Lactobacillus fermentum* C14) will be pronounced.

N. C Mandal
30.12.13

Narayan C. Mandal, Ph.D
Professor of Botany
Visva-Bharati
Santiniketan-731235
