# Supplementary material for: Longterm storage of post-packaged bread by controlling spoilage pathogens using Lactobacillus fermentum C14 isolated from homemade curd
Source: PLoS One. 2017 Aug 31;12(8):e0184020. doi: 10.1371/journal.pone.0184020 (PMC5578496; doi:10.1371/journal.pone.0184020)
Supplement: S3 File — (PDF) [file pone.0184020.s003.pdf]

To
The Chairman
Animal Ethics Committee
Visva-Bharati

30.12.2013

Subject: Permission to carry out animal experiment for our research

Respected Madam,

I would like to perform one animal experiment to check any kind of toxicity by *Lactobacillus fermentum* C14 in mammalian system (Swiss albino mice). Bacterial suspension will be administered to Swiss albino mice for a month through drinking water in respective feeding bottles and toxicity will be measured by assaying liver enzymes. The detail of the experiment is attached herewith.

Please do the needful for your kind approval.

Yours sincerely

N. C. Mandal
30.12.13

Narayan C. Mandal , Ph.D.
Professor of Botany
Department of Botany
Visva-Bharati
Santiniketan-731235

The proposal of research is approved since non pathogenic bacteria only will be used.
Shelley Bhattacharya
Chairperson. 24/01/2014

Chairperson
Animal Ethics Committee
Visva-Bharati
Santiniketan, West Bengal
